# Evaluating fisheries conservation strategies in the socio-ecological system: A grid-based dynamic model to link spatial conservation prioritization tools with tactical fisheries management

**DOI:** 10.1371/journal.pone.0230946

**Published:** 2020-04-03

**Authors:** Yunzhou Li, Ming Sun, Chongliang Zhang, Yunlei Zhang, Binduo Xu, Yiping Ren, Yong Chen

**Affiliations:** 1 College of Fisheries, Ocean University of China, Qingdao, China; 2 School of Marine Sciences, University of Maine, Orono, Maine, United States of America; 3 Laboratory for Marine Fisheries Science and Food Production Processes, Pilot National Laboratory for Marine Science and Technology (Qingdao), Qingdao, China; Swedish University of Agricultural Sciences and Swedish Institute for the Marine Environment, University of Gothenburg, SWEDEN

## Abstract

Spatial conservation prioritization concerns trade-offs between marine conservation and resource exploitation. This approach has been increasingly used to devise spatial management strategies for fisheries because of its simplicity in the optimization model and less data requirement compared to complex dynamic models. However, most of the prioritization is based on static models or algorithms; whose solutions need to be evaluated in a dynamic approach, considering the high uncertainty and opportunity costs associated with their implementation. We developed a framework that integrates species distribution models, spatial conservation prioritization tools and a general grid-based dynamic model (Grid-DM) to support evaluation of ecological and economic trade-offs of candidate conservation plans. The Grid-DM is spatially explicit and has a tactical management focus on single species. We applied the Grid-DM to small yellow croaker (*Larimichthys polyactis*) in Haizhou Bay, China and validated its spatial and temporal performances against historical observations. It was linked to a spatial conservation prioritization tool Marxan to illustrate how the model can be used for conservation strategy evaluation. The simulation model demonstrated effectiveness in capturing the spatio-temporal dynamics of the target fishery as well as the socio-ecological effects of conservation measures. We conclude that the model has the capability and flexibility to address various forms of uncertainties, simulate the dynamics of a targeted fishery, and to evaluate biological and socioeconomic impacts of management plans. The modelling platform can further inform scientists and policy makers of management alternatives screening and adaptive conservation planning.

## 1. Introduction

Overexploitation of marine fisheries remains a serious issue across the globe. Even for fisheries that have been intensively managed by coastal nations, various sources of uncertainty have greatly impeded the effectiveness of traditional fisheries management approaches in replenishing exploited stocks [1]. Spatial management, especially marine protected areas (MPAs), are quickly receiving popularity as a precautionary approach to assure the sustainability of populations and guard against fisheries failures [1–4]. However, many practical issues arise in the design of spatial management, requiring sound knowledge of ecological processes and population dynamics. The planning process becomes more deterring when social and economic objectives are integrated into a planning framework in addition to the ecological objectives, requesting for more sophisticated trade-off analysis in the decision-making process [5]. Two commonly-used objectives for fisheries management and conservation are to effectively allocate conservation actions to minimize socio-economic impacts on local fishing communities and support socio-economic development that minimizes conservation impacts. Herein, decisions need to be made with a full understanding of trade-offs between marine conservation and resource exploitation.

A range of decision-support tools have been developed to guide the design of spatial conservation strategies for fisheries management, including Marxan [6] and Zonation [7]. In general, these tools adopt a grid-based approach that divides the planning region into a certain size of grid planning units (PUs), assigns ecological attributes, human activities, and socioeconomic data among PUs, and identifies what areas should be protected to achieve a desirable conservation goal at the minimum socioeconomic cost. Here, PUs are defined as a collection of cells from which a conservation planner selects new conservation areas. Over the past decade, these spatial conservation prioritization tools received increasing attention because of their simplicity in the optimization approach and less data requirement compared to complex dynamic models [8]. Many applications suggest that spatial conservation prioritization tools can effectively optimize MPA designs to balance ecological and economic objectives for fisheries management [9–10]. Nevertheless, most of the spatial conservation prioritization is based on static models or algorithms whose solutions need to be evaluated in a dynamic approach, considering the high uncertainty and opportunity costs associated with their implementation. In this regard, it is necessary to develop a dynamic evaluation approach to better inform potential socio-ecological consequences of spatial management solutions derived from grid-based conservation planning tools, and support policy screening, evaluation and identification for fisheries management and conservation.

Evaluation of spatial fisheries management can be achieved at various operational resolution levels. The simplest simulation models of the lowest resolution are two-patch population models that divide the ecosystem into two patches: fishing ground and protected area. They are mainly used to investigate the effect of no-take MPA size on fisheries, but are critiqued for their limited ability in simulating spatial dynamics of populations and assessing the effects of multi-zone MPAs [11]. White et al. [12] presented a one-dimensional spatially explicit model to model fish population dynamics along the California coastline. Rassweiler et al. [13] developed a more advanced spatially explicit model to evaluate the spatially optimized fisheries management. Despite their advantage in examining the spatio-temporal dynamics of a single species, the resolutions of the models are limited by available habitat patches, and thus lack flexibility in dealing with management solution evaluation in a broad grid-based planning context. By contrast, grid- or polygon-based ecological models, such as Ecospace [14], OSMOSE [15], and Atlantis [16], provide a general approach to investigate community, ecosystem, or socioeconomic effects of MPAs (for example, [17–19]). However, these models have a high requirement for both data and computer processing capacity. Additionally, both results and uncertainties from ecosystem models can be too complex to be interpreted for tactic fisheries management [11]. For instance, these models may not be able to reflect the detailed dynamics of a specific stock in the near term or in specific geographical locations [20]. Therefore, the need emerges to develop a less-data-needy grid-based spatially explicit dynamic model that can contribute to a structured evaluation of spatial conservation prioritization solutions for fisheries management and conservation.

This study develops a framework to support the evaluation of fisheries conservation strategies in the socio-ecological system. Specifically, we present the structure of a grid-based dynamic model (Grid-DM) that links spatial conservation prioritization tools with tactical fisheries management at the single species level. The developed framework is then implemented to small yellow croaker (*Larimichthys polyactis*) inhabiting Haizhou Bay, a typical fishing ground and conservation hotspot in China, as a case study. We establish the model to simulate the spatio-temporal dynamics of the target fishery with existing fisheries conservation measures. We then link the validated model to the spatial conservation prioritization tool, Marxan, to illustrate how the proposed framework can be used for conservation strategy evaluation. By bridging conservation planning and fisheries management, this modeling framework can inform scientists and policy makers on the socio-ecological effects of spatial management measures and using spatial conservation prioritization to support the identification of best management policies for their fishery.

## 2. Materials and methods

### 2.1. A framework to support evaluation of spatial conservation prioritization solutions for fisheries management

**Fig 1:**
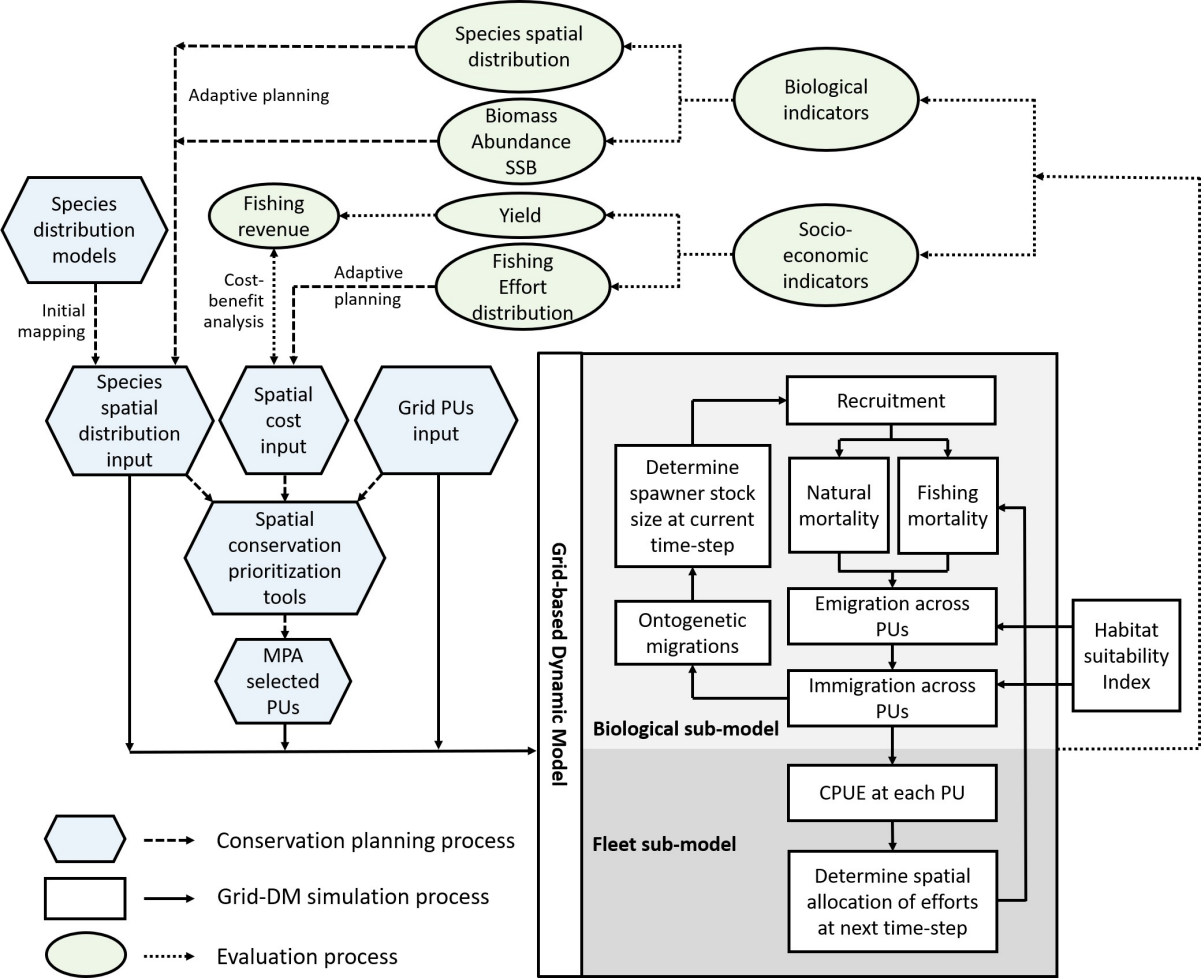
Conceptual framework of grid-based dynamic model (Grid-DM) for evaluation of spatial planning solutions.

We developed a framework that links spatial conservation prioritization tools with tactical fisheries management, contributing to a systematic and adaptive decision-making process for conservation planning in the realm of fisheries (Fig 1). The framework primarily consists of three major components, including 1) the conservation planning component with the support of spatial conservation prioritization tools, 2) the simulation component using a 2D spatially explicit dynamical model, and 3) the evaluation component based on performance metrics. These components link to each other through multiple data layers and constitute a closed feedback loop for developing adaptive conservation measures. For instance, maps of species distribution and habitat quality can be used in spatial prioritization tools, such as Marxan, along with other socio-economic information. In addition, this species distribution map can be used as initial distribution and baseline information for simulation and evaluation. The habitat quality map can be used to parameterize or calibrate movement rates between adjacent patches in the spatially explicit mechanistic fish population dynamic models, as they can be an important driver of fish movement and distribution. Furthermore, the dynamic fisheries model can be used to assess the fishery and conservation performance of existing or alternative MPA networks proposed by using spatial conservation prioritization tools. Lastly, the predicted species distribution from the dynamic model can be further applied in the conservation planning process to advise adaptive planning.

The conservation planning process relies on three key input datasets (Fig 1). These datasets include Grid PUs input, species spatial distribution input, and spatial cost input. Specifically, the PU grid file can be created by ArcGIS extension ET Geowizards (http://www.ian-ko.com/). This grid file includes a PU identification number and location information. Species distribution models (SDMs, [21]) offer a great potential for marine conservation and planning. SDMs identify relationships between the geographical distribution of species and characteristics of environmental factors [22]. Such models can predict species’ habitat suitability and species’ probability of occurrence based on environmental variables. Considered as useful tools that offer a viable compromise between the lack of comprehensive species distribution data and the expense of collecting addition data, SDMs are increasingly utilized in conservation planning as an approximation to species ranges [23–26]. Accordingly, we use species distribution maps derived from SDMs as an input layer for MPA designation, according to previous studies [23–26]. The spatial cost input defines the cost value for each PU. The choice of cost data usually depends on the socio-economic objectives of MPAs, which can be either a simple reflection of PU area or potential loss in fishery yield due to conservation, corresponding to the objectives that minimize MPA size or minimize harvest loss, respectively [27]. With these inputs, spatial conservation prioritization tools, such as Marxan and Marxan with Zones, generate outputs that identify where to place MPAs (no-take reserves or multi-zone MPAs that allow sustainable uses in certain zones). In our proposed framework, the inputs (e.g., species distribution data, spatial cost data, and grid PU information) and outputs (e.g., PUs selected as an MPA) of prioritization tools can be directly used in the Grid-DM (See Section 2.2) with their original formats as a premise for simulation. Specifically, Grid-DM reads the PU information in the Grid PU file and constructs the two-dimensional base grid for simulation. Second, Grid-DM adopts the species spatial distribution input as the initial distribution of target species. It should be noted that conservation prioritization tools may be run for conserving multiple types of biodiversity, using multiple input layers of species distribution. As our framework is primarily focused on evaluating tactical management measures for a single species, a single species distribution input can be utilized in the Grid-DM to investigate the effect of optimized MPA solutions for the biodiversity conservation objective on a specific concerned species, e.g., the most conservation needy species. Third, Grid-DM overlays the output file of PUs selected as no-take reserves or PUs zoned with restrictions on fishing gears over the grid PUs and species distribution layers. This further defines the allocation of fishing efforts over PUs. By using these inputs and outputs from the conservation prioritization tools into the simulation model, this approach not only reduces the data requirement for the Grid-DM, but also ensures data consistency throughout the conservation planning and evaluation processes.

Hexagons in light blue and dashed lines indicate the conservation planning process. Rectangles in white and solid lines indicate the Grid-DM simulation process. Ovals in light green and dotted lines indicate the evaluation process. Lighter gray represents components of biological sub-model; darker gray represents components of fleet sub-model. PU: planning unit; MPA: marine protected area; CPUE: catch per unit effort; SSB: spawning stock biomass.

As the framework aims to provide assessments for fisheries conservation solutions, we developed a set of evaluation metrics to represent their biological and socio-economic performances (Fig 1). The biological indicators reflect the stock’s biological status with different management measures. These indicators include biomass, abundance, and spawning stock biomass. During the simulation, the species spatial distribution is subject to habitat-quality-related fish movement patterns, spatial management measures, and harvest from fishery. In the presented framework, this output can, in turn, provide feedback to the prioritization tools and support adaptive planning and management. The adaptive planning then refines spatial management plans for establishing new premises for the future simulation. Given a wide application of spatial conservation prioritization tools to minimize socio-economic impacts of MPAs on fisheries, we additionally develop socio-economic indicators to illustrate the medium/long-term socio-economic effects of conservation planning solutions. This complements the short-term socio-economic cost estimated by Marxan and Marxan with Zones, which assumes the cost to be fixed after the MPA implementation without the reallocation of fishing effort [28]. These socio-economic indicators can be linked to the prioritization tools by two means (Fig 1). First, the model evaluates potential fishing revenue based on predicted yield and supports cost-benefit analysis for policy screening. The cost could be potential loss in fishing revenues due to conservation. Second, the model simulates fleet dynamics and predicts fishing effort distribution at next time step; this output can be further applied, along with the predicted fishing revenue information, as a cost layer in prioritization tools for adaptive planning.

### 2.2. A grid-based dynamic model

#### 2.2.1. Overview

Grid-DM includes two components: a biological sub-model and a fleet sub-model (Fig 1). The spatial dynamics of fish populations and fleet are represented over grid PUs derived from spatial conservation prioritization tools. A monthly time step is used to describe seasonal patterns, considering the seasonality in both fish populations (i.e., seasonal migrations) and management strategies (i.e., seasonal closure).

#### 2.2.2. Biological sub-model

The biological sub-model simulates the spatio-temporal dynamics of the fish stock, using age-structured data with spatial attributes. Within each time step and PU, biological processes occur in the following order: (i) recruitment in spawning seasons, (ii) population decrease caused by natural mortality and harvest, (iii) emigration to adjacent PUs, and (iv) immigration from adjacent PUs. In addition, the biological sub-model considers effects of ontogenetic migrations, such as spawning and wintering migrations, at a larger scale. These migrations are represented as a seasonal flux in the biological sub-model depending on the migration behaviors of the target stock.

**Fig 2:**
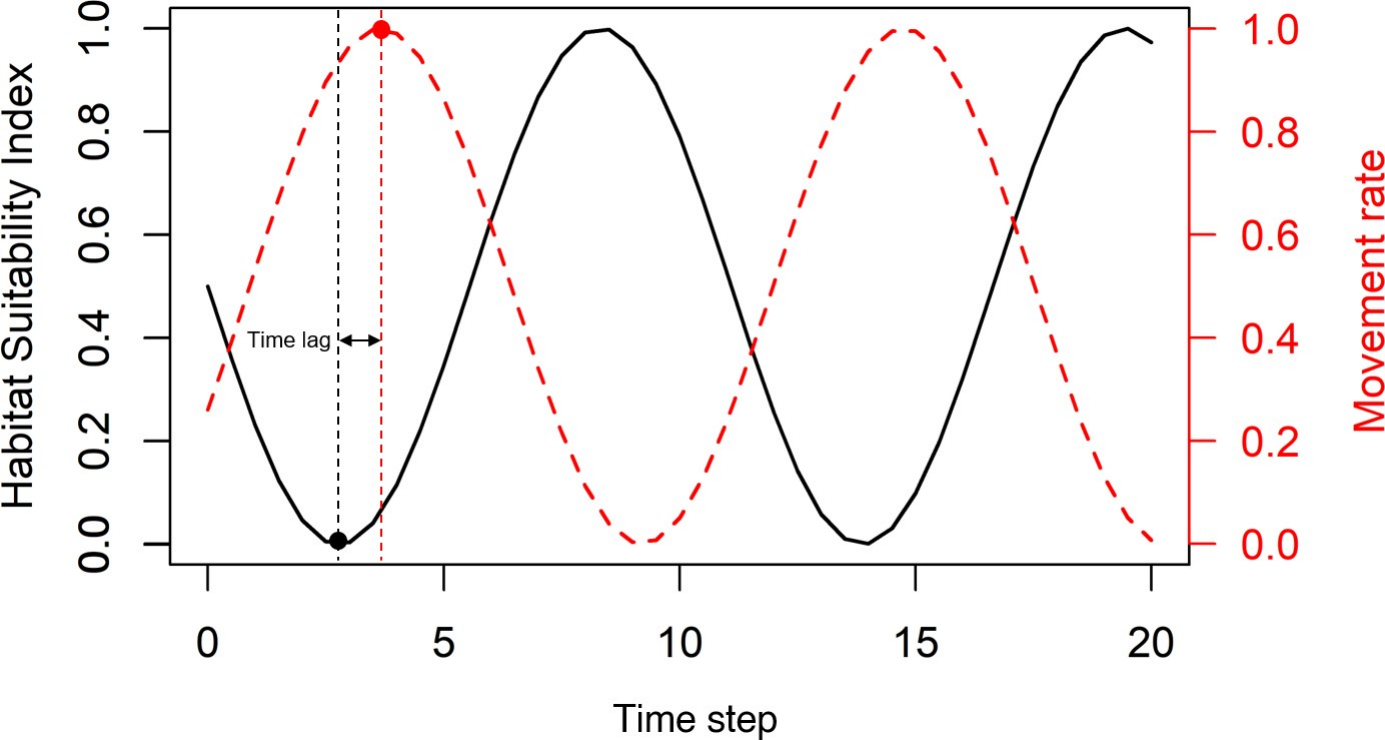
The calculation of movement rate based on habitat suitability index (HSI) for each PU. The first step is to fit a sinusoidal function of timesteps by the seasonal HSI data for each PU. Then the movement rate curve is the reflected HSI curve over the line y = 0.5. Considering the time lag in fish response to the habitat suitability [33], the movement rate curve has a horizontal shift.

Specifically, recruitment models would be developed depending on target species. For instance, Beverton-Holt model can be used to simulate density-dependent recruitment occurring within a PU:

$$\begin{array}{l} R_{i,t}=\left(\frac{\alpha S_{i,t-1}}{1+\beta S_{i,t-1}}\right)e^{\varepsilon },\in \left\{\mathrm{time}\;\mathrm{steps}\;\mathrm{when}\;\mathrm{spawning}\;\mathrm{occurs}\right\},\\ \varepsilon \in \{\mathrm{sampled}\;\mathrm{residuals}\}, \end{array}$$ (1)

where *R*_*i*,*t*_ indicates the abundance of recruits at time step *t* and the *i*^th^ PU. *S*_*i*,*t*−1_ denotes spawning biomass at time step *t*-1 and the *i*^th^ PU. ε refers to the residual of spawner-recruitment relationship (SRR). Parameter *α* is a density-independent parameter proportional to fecundity, and *β* is a density-dependent parameter proportional to both fecundity and density-dependent mortality. Species abundance subject to mortality was calculated before movement:

$${preN}_{a,i,t}=N_{a,i,(t-1)}e^{-(F_{i,t}{Sel}_{a}+M)},$$ (2)

where *preN*_*a*,*i*,*t*_ indicates pre-movement abundance at age *a*, time step *t* and the *i*^th^ PU. *N*_*a*,*i*,(*t*−1)_ refers to abundance at age *a* and the *i*^th^ PU in the previous month. *M* is natural mortality. *F*_*i*,*t*_ is fishing mortality at full selectivity at time step *t* and the *i*^th^ PU, which is calculated in the fleet sub-model. *Sel*_*a*_ is selectivity at age *a*. Fish movement is allowed across the borders to four adjacent PUs. At each time step, a fraction of individuals moves to and distributes evenly in the adjacent PUs [29]. The probability of fish movement from each PU is assumed to be affected by two parameters: one is basic movement rate (*m*) driven by the habitat suitability of each PU for the targeted stock, and the other is movement rate coefficient (*ω*) that defines the general movement ability of the stock within the study area. Both were scaled between 0–1. Specifically, *m* is assumed to bea function of habitat suitability index (HSI) [30] with a time lag within each PU (See Fig 2 for details), assuming that HSI in each PU has seasonal patterns [31] and that the probability of fish movement from each PU is negatively related to the HSI value in each PU [32]. For instance, PU with a high habitat suitability index indicates higher retention, which translates into low basic movement rates in the next time step in the model. Considering the variability in movement ability of different stocks, the movement rate coefficient parameter needs to be identified for ad hoc stocks and resolutions. For each PU, the abundance after movement (*postN*_*a*,*i*,*t*_) was calculated as:

$$postN_{a,i,t}=preN_{a,i,t}-{\omega m}_{i,t}N_{a,i,t}+\sum_{j}^{n}{{\omega m}_{j,t}N}_{a,j,t},$$ (3)

where *m*_*i*_ and *m*_*j*_ denote basic movement rate at the *i*^th^ PU and the adjacent *j*^th^ PU respectively, varying by HSI across PUs. *n* indicates the sum number of adjacent PUs of the *i*^th^ PU. *ω is* movement rate coefficient and is constant.

#### 2.2.3. Fleet sub-model

The fleet sub-model aims to simulate the dynamics of fleet and determine the allocation of fishing effort, which further defines the fishing mortality rate in each PU in the biological sub-model. Different gear types can be simulated as different fleets with the sub-model. Accordingly, the model allows applications to multi-zone MPAs that have different spatial restrictions of gear uses in multiple zones. Here, the dynamics of single fleet was presented.

The fleet sub-model is modified from Rassweiler et al. [13] and Brown et al. [34] by incorporating catch per unit effort (CPUE) as the driver of fleet aggregation. Like other fleet models, this sub-model assumes a fixed total fishing effort within each year [35]. The underlying assumption is that fishers tend to aggregate in the area with high CPUE. The best knowledge of fishing effort distribution would have to be acquired from the CPUE from the precedent time step, indicating a minor lag is inevitable [13]. Accordingly, total fishing efforts are spatially allocated among PUs based on their corresponding CPUE. Considering the imperfection in fishers’ knowledge of CPUE, the model considers a fleet aggregation factor (*v*) that reflects fishers’ knowledge on CPUE, for example, a larger *v* represents fishers’ better knowledge of CPUE and thusly contributes to more clustered fishing efforts in high CPUE area (or more significant overlap of them). The catch, CPUE and fishing mortality at time step *t* and *t*+1 in the *i*^th^ PU are calculated with equations modified from Rassweiler et al. [13] and Brown et al. [34]:

$$C_{i,t}=\sum_{a=1}^{A}\frac{F_{i,t}{Sel}_{a}}{F_{i,t}{Sel}_{a}+M}\left(1-e^{-(F_{i,t}{Sel}_{a}+M)}\right)N_{a,i,t}W_{a},$$ (4)

$${CPUE}_{i,t}=\frac{C_{i,t}}{F_{i,t}},$$ (5)

$$F_{i,t+1}=F_{T}({\left({CPUE}_{i,t}x_{i,t}\right)}^{v}/\sum_{i=1}^{nPU}{\left({CPUE}_{i,t}x_{i,t}\right)}^{v}),$$ (6)

where *C*_*i*,*t*_ is catch at time step *t* and the *i*^th^ PU, *F*_*i*,*t*_ indicates fishing mortality at time step *t* and the *i*^th^ PU. *W*_*a*_ is weight at age a. *A* is the plus age class. *F*_*T*_ is the total effort. *x*_*i*,*t*_ is a Boolean variable indicating whether the *i*^th^ PU is open to fishing at time step *t* with 1 as true and 0 as false. *v* is the fleet aggregation factor. *nPU* is the sum number of PUs. Effort compensation is included when there is seasonal or spatial closure to ensure the total fishing effort remains the same under different management measures [34]:

$$F_{T}=F_{base}\left(1+\frac{n_{c}}{n_{o}}\right)\left(1+\frac{{PU}_{c}}{{PU}_{o}}\right),$$ (7)

where *F*_*base*_ indicates total fishing effort before a management approach is implemented, *n*_*c*_ and *n*_*o*_ indicate the number of closed and open months in a year for fishing, respectively. *PU*_*c*_ and *PU*_*o*_ indicate the number of closed and open PUs in a year for fishing, respectively.

### 2.3. A case study in Haizhou Bay, China

#### 2.3.1. Study area and target fish

We applied the proposed Grid-DM to small yellow croaker (*Larimichthys polyactis*) in Haizhou Bay, (119°11′- 122°00′ E, 34°00′- 35°50′ N), which is an important fishing ground in China. This stock used to support a major fishery in China and Haizhou Bay. However, the stock has been substantially exploited since the 1970s and is considered overfished [36]. The small yellow croaker stock in Haizhou Bay experiences two major migrations in its life history: one is the spawning migrations to Haizhou Bay before its spawning season in May and the other is the wintering migrations offshore in November [37–38]. We collected biomass data from a bottom trawl survey conducted in September 2011 and 2013–2017, permitted by the Ministry of Agriculture of People’s Republic China and Ocean University of China (Supporting information). A stratified random sampling design was used with a total of 24 sampling sites in 2011 and 18 sites in the following years being selected from five strata per survey [39]. The bottom trawl was towed at a speed of 2–3 knots for about 1 h, with a trawl net of 12m width and mesh size of 17mm. The two vessels used in the bottom trawl survey have the same size, power and are using the same net and tow speed. At each survey station, a CTD system (XR-420) was used to measure environmental data including depth, bottom temperature and bottom salinity.

**Fig 3:**
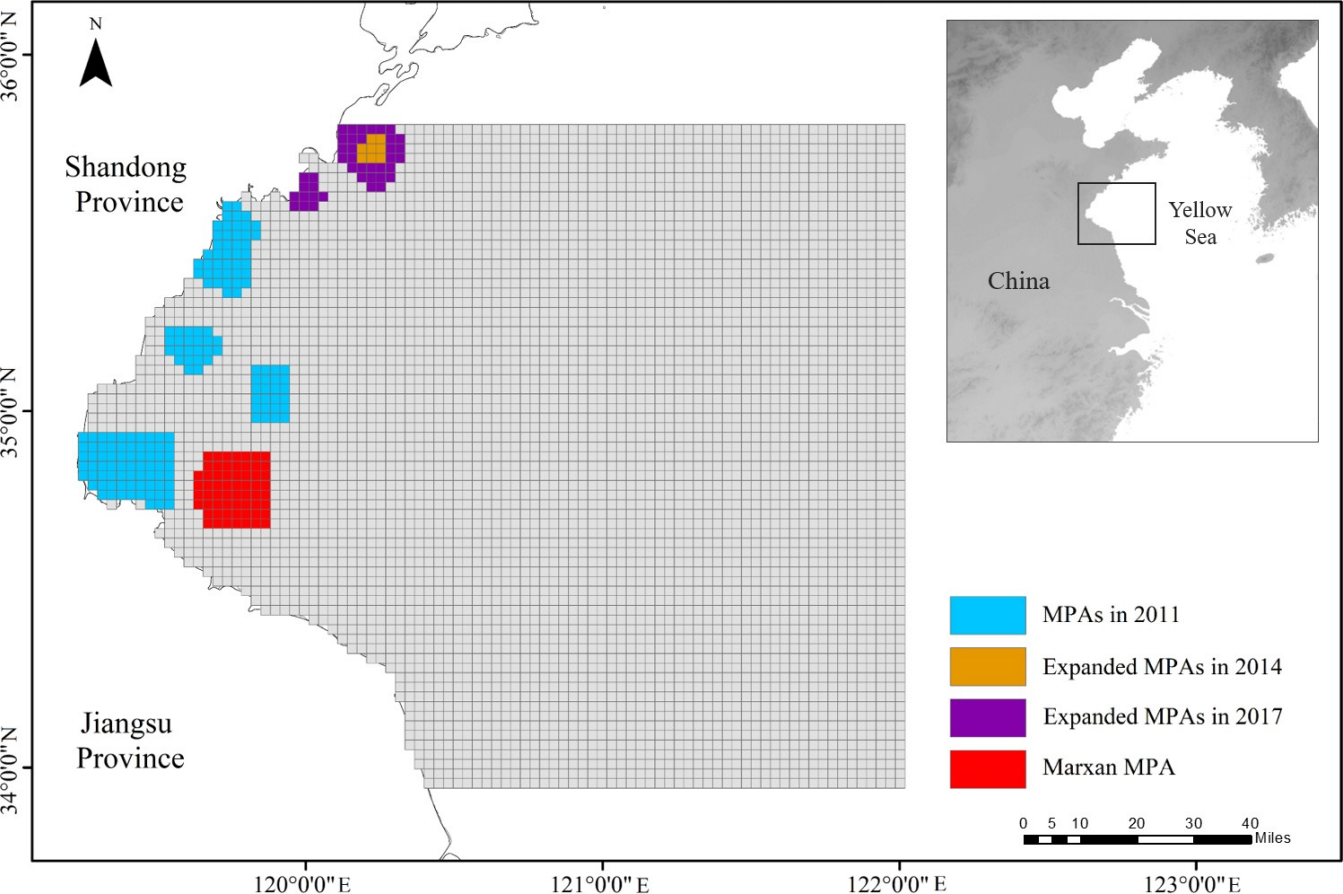
Spatial configuration of existing MPAs and Marxan-derived alternative MPA. Existing MPAs include MPAs in 2011 and additional MPAs expanded respectively in 2014 and 2017.

We divided the study area into 4815 square planning units (PUs) of 9 km^2^ (Fig 3), based on the scale of the study area, existing MPAs, and historical observations of the species movement [37–38]. The species distribution in September 2011, and 2013–2017 was predicted using generalized additive model (GAM) [40] and Finite Volume Community Ocean Model (FVCOM) [41], and was then interpolated using inverse distance weighting (IDW) tool in ArcGIS 10.2 [42] for MPA prioritization and simulation (S1 Fig) (S1 Material).

#### 2.3.2. Model parameterization

To reflect the species ontogenetic characteristics, a seasonal influx of spawners before recruitment in May and an outflux of all age-classes stocks in November are included in the biological sub-model, wherein the flux rate was estimated based on Zhong et al. [38] (Table 1). A density-modified SRR [43] was developed to describe recruitment of this stock (Table 1) (Eq 1). Specifically, the original absolute SSB and recruits were converted into density values by dividing them with the area of the region. The obtained density of SSB and recruits were then used as variables to estimate the modified SRR. Natural mortality and fishing mortality were estimated with length-frequency data for the stock using survey data from 2011 and 2013–2017 (Table 1) [44]. While the parameters described above were derived from the survey data in 2011 and 2013–2017, the parameter values of 2012 were assumed to be the average of those in 2011 and 2013 due to the lack of data. Basic movement rate for each PU was modeled based on habitat preference measured with HSI (S1 Material; S1 Table; S2 and S3 Figs).

**Table 1:** Summary of parameters used in the model. LN(0,0.2) indicates a lognormal distribution of error with mean of 0 and standard deviation of 0.2.

Parameters	Values							Reference
	2011	2012	2013	2014	2015	2016	2017	
Natural mortality, M (year^-1^)	0.52	0.49	0.45	0.52	0.37	0.48	0.48	[44]
Fishing mortality, F (year^-1^)	0.72	0.89	1.06	0.65	0.41	0.52	0.74	[44]
Linear growth parameter at low stock size, *α*	0.14							[43]
Density-dependent parameter, *β*	6.674*10^−5^							[43]
Seasonal influx rate (%)	61.33							[38]
Seasonal outflux rate (%)	53.51							[38]
price, *μ* (dollar per kilogram)	2.85							Unpublished data
Discount rate, *r* (year^-1^)	0.1							Assumed
Recruitment error	LN(0,0.2)							Assumed
Movement rate error	LN(0,0.2)							Assumed
Fleet aggregation error	LN(0,0.2)							Assumed

#### 2.3.3. Calibration

Movement rate coefficient *ω* and fleet aggregation factor *v* were two vital parameters in quantifying the spatial dynamics of both the stock and fleet. Their parameterizations could be preliminarily determined to an approximate range of values, which needed to be further calibrated at the case-specific level. The calibration procedure was performed at PU and year levels with hindcast deterministic simulation, where all knowledge during the historical years was available in a hindsight. Calibration runs were conducted to obtain the optimal value of *ω* and *v* that could explain the spatio-temporal dynamics of stock from 2011 to 2017. The iterative least-square approach was adopted in the calibration procedure by iteratively approximating the optimal parameter value that minimized the sum of squared differences between observations and simulations [45]. The species distribution layer of 2011 was used as an initial distribution dataset and a baseline for simulation (See Section 2.3.1), those of other historical years (species distribution layers of 2013–2017) were used for calibrating criteria as observed data (See Section 2.3.1). Specifically, both the observed biomass and simulated biomass in September, 2013–2017 in each PU were transformed to Δ-distribution for comparison [46]:

$$standardized\;biomass=\frac{\ln\left(biomass\right)-\bar{\ln\left(biomass\right)}}{sd(\ln\left(biomass\right))},$$ (8)

#### 2.3.4. Spatial conservation prioritization

Existing MPAs were mostly designed in coastal areas with less important commercial values and higher enforcement capacity [47]. Unlike this approach, we used spatial conservation prioritization tool Marxan [6] to identify an alternative MPA and test whether the Marxan-derived MPA would outperform the existing MPAs in biological and socio-economic performances. Marxan was developed to find a range of solutions that meet the protection target while minimizing the cost [48]. The protection target was defined as the amount of the conservation species to be included within the MPA (i.e., percentage of species’ biomass), while the cost can be calculated either as simple reflection of PU area, or as an economic cost such as potential loss in fishing revenue due to conservation [27]. In this case study, instead of designing a complex and perfect management scenario, we focused on demonstrating how the outputs from spatial optimization tools could be used for the simulation of Grid-DM with a simple exemplary scenario. We adopted the percentage of total biomass of small yellow croaker included within the existing MPAs in 2011 as the protection target (3.65%) and considered PU area as the cost to select an alternative MPA that offers the same protection level as the existing MPAs with a minimal MPA size (S1 Material). 4815 square PUs of 9 km^2^ and the spatial distribution of stock biomass in 2011 were used as the import layer (same as Section 2.3.1). According to Ardron et al. [27], Marxan was run for 100 times, where the best solution output was adopted as an alternative MPA and was further evaluated by the Grid-DM (S1 Material).

#### 2.3.5. Evaluation of fisheries conservation strategies

We incorporated the existing management measures in our simulation for model validation, including seasonal closure and/or spatial closure restricted by MPAs. Seasonal closure is an important fisheries management measure in China that restricts fishing activities during summer [49]. In addition, MPAs were designated in Haizhou Bay to limit extractive uses for the purpose of marine resources conservation. We simulated both temporal and spatial changes in China’s fisheries management and conservation policy from 2011 to 2017 (Table 2). For instance, the national seasonal closure policy was extended from a three-month closure (from June 1^st^ to August 31^st^) to a four-month closure (from May 1^st^ to August 31^st^) in 2017; MPAs in Haizhou Bay were expanded in 2014 and 2017 (Fig 3).

**Table 2:** Temporal and spatial changes of management measures in 2011–2017.

Management measures	2011	2012	2013	2014	2015	2016	2017	Marxan-selected MPA
Seasonal closure	June—August	June—August	June—August	June—August	June—August	June—August	May—August	Same as hindcast simulations
MPA*	158	158	158	167	167	167	209	60

* Number of planning units selected as marine protected area (MPA)

The Grid-DM was further run for 7 years (2011–2017) to simulate the temporal and spatial dynamics of the targeted fishery. We additionally incorporated stochasticity in the simulation to represent multiple sources of uncertainties in the population dynamics and fisheries that might render the management ineffective. These uncertainties include the fluctuations in recruitment, variations in fish movement and imperfect knowledge of fisherman to locate a productive fishing ground (**Table 1**). These variations were added to the SRR estimation and calibrated movement rate as well as fleet aggregation factor, respectively. To understand the robustness and accuracy of Grid-DM in demonstrating the existing management measures’ impacts on the stock, we validated the simulated results against the observations with both temporal and spatial metrics. Specifically, we (i) compared the temporal changes in simulated biomass to the observed biomass over the study area; (ii) compared the spatial variation in simulated biomass with observed distribution at a PU level. Both simulated and observed biomass in each PU were standardized for comparison (Eq 8) (See Section 2.3.3).

We additionally simulated the Marxan-derived MPA to illustrate the use of Grid-DM for conservation planning solution evaluation. MPA was considered as no-take reserves throughout the simulation. The best solution output from Marxan was directly imported to the Grid-DM, with its original binary format, and then was overlaid to the PU layer to define the allocation of fishing effort (Eq 6). Configuration of parameters and stochasticity were identical to that for the hindsight simulation. For this comparison, we additionally conducted a preliminary cost-benefit analysis for the two MPA scenarios. We considered fishing revenue from harvesting as the benefit and the establishment and maintenance costs as the cost. Both benefits and costs were discounted to the present value for analysis. The present fishing revenue (R) was calculated as:

$$R=\sum_{t=1}^{T}\frac{\mu C_{t}}{{\left(1+\frac{r}{12}\right)}^{t}},$$ (9)

where *C*_*t*_ is the total catch at time step *t*; *μ* is the price of small yellow croaker; *r* is the discount rate; *T* is the last time step. The present establishment cost (EC) and maintenance cost (MC) were calculated with Eqs (10) and (11) from McCrea-Strub et al. [50], respectively, which were based on representative data gathered from global MPA literature:

$$EC=10^{4.66}a^{0.52},$$ (10)

$$MC=10^{5.23}a^{0.21}y,$$ (11)

where *a* is the area of MPA and *y* is the implementation year of MPA. The open source programming language R (version 3.4.0) was used in this study [51]. Visualization of the simulation results was achieved with R package “ggplot2” (version 2.2.1) [52] and ArcGIS 10.2 [42].

## 3. Results

**Fig 4:**
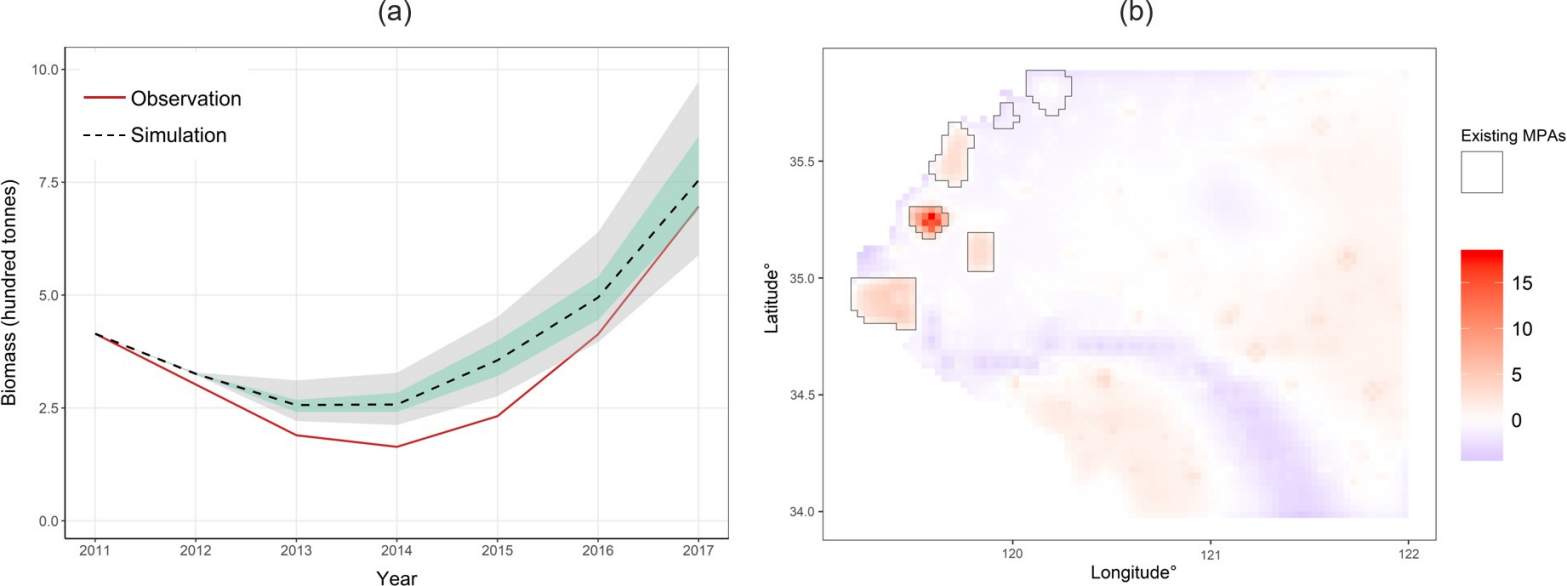
Comparison between simulation and observation for the month of September in (a) temporal biomass change from 2011 to 2017 and (b) species spatial distribution in 2017. (a): areas shaded in grey and green indicated 95% and 50% quantile respectively; dashed line indicates the median biomass. (b): standardized median biomass distribution from simulations and standardized biomass distribution from observations were compared in 2017. Darker red represents higher standardized median biomass in simulation than observation (See S5 Fig and S6 Fig for full figures).

The movement rate coefficient *ω* of 0.24 and a fleet aggregation factor *v* of 1.13 were used in the simulation based on calibration (S4 Fig). We analyzed the performance of the simulation model based on temporal and spatial comparisons between simulations and observations in September. For temporal biomass trend, the simulated biomass trend resembled the observed trend well; both showed a declining trend before 2014 and an upward trend afterwards (Fig 4A). However, it was observed that simulation had a slower declining rate compared to that with observation in the beginning years. During 2016 and 2017, both had a similar increasing rate, which was the highest among the increasing rates for all the simulated years. In addition, results of stochastic runs were illustrated in Fig 4A. The model showed fluctuations on the biomass with consideration of variations in population and fleet dynamics. The observed biomass was also within the 95% quantile of stochastic simulations during 2016 and 2017. The evaluation of spatial performance of the model is based on the comparison of species spatial distribution from observations and simulations in 2017 (Fig 4B). The simulated biomass distribution was fairly consistent with the observations in most areas. Interestingly, we found an overestimation of biomass inside several existing MPAs. Overall, simulations and observations had a moderate correlation (*r*^2^ = 0.43, 95% confidence level of *r*^2^ = 0.42).

**Fig 5:**
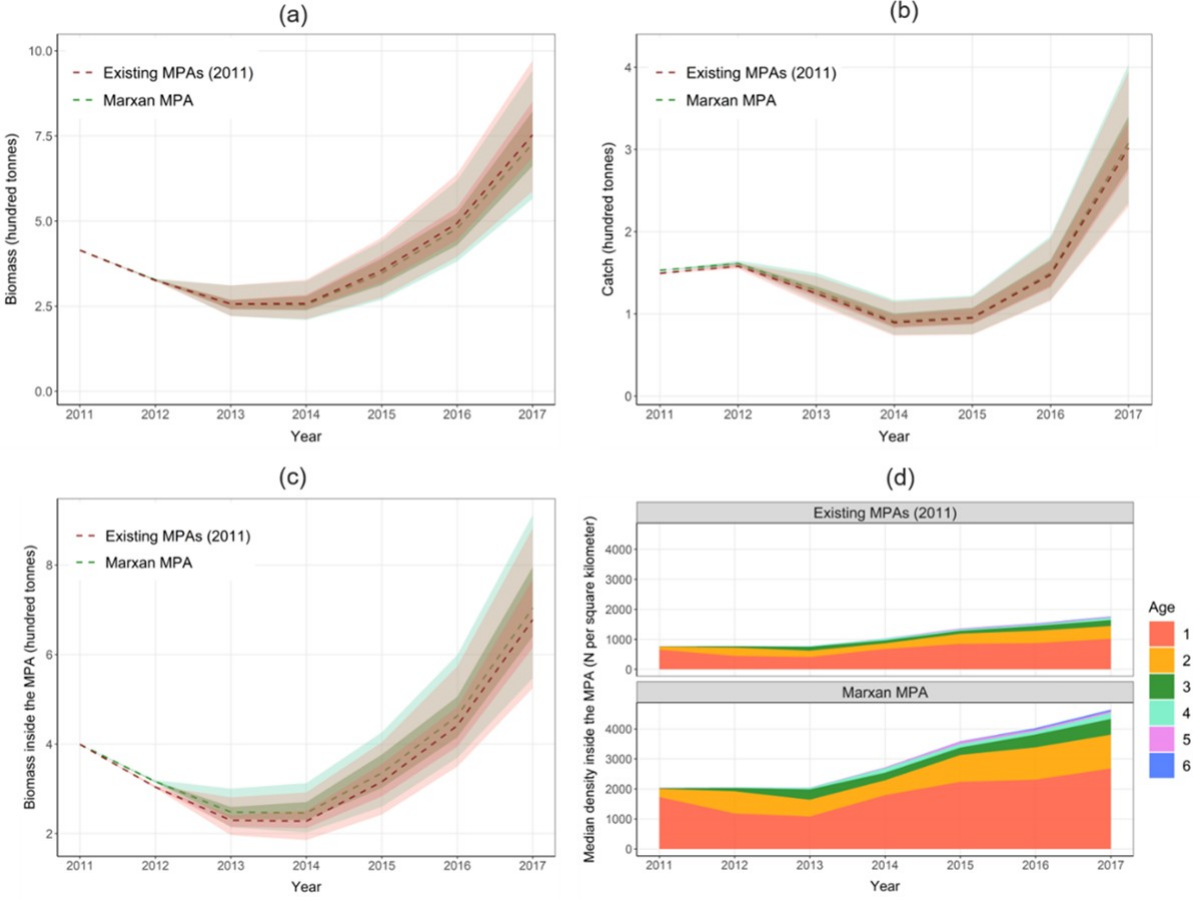
Simulation results of implementing existing MPAs in 2011 and Marxan-derived alternative MPA: (a) total biomass; (b) total catch; (c) biomass inside the MPA; and (d) Median fish density by age class inside the MPA. The simulated biomass and catch from two MPA scenarios were illustrated with median (dashed line), 95% quantile (light green for Marxan MPA and light pink for Existing MPAs), and 50% quantile (dark green for Marxan MPA and dark pink for Existing MPAs).

With the identical protection target for the existing MPAs in 2011 and additional socio-economic objective to minimize the MPA area and associated management costs, Marxan identified an MPA as an alternative spatial management strategy (Fig 3). This alternative MPA was located in the coastal area, adjacent to the existing MPAs. The size of MPA was significantly reduced to nearly 40% of that of existing MPAs in 2011 (Table 2). However, we found that this smaller-size alternative MPA had nearly 4% lower of median total biomass and 2% higher of median total catch compared to those with the existing MPAs scenario (Fig 5A and 5B). Furthermore, the alternative MPA had more biomass inside the MPA than the existing MPAs at the end of simulation, despite that both had the same biomass inside the MPA initially (Fig 5C). A higher fish density was also observed inside the alternative MPA than that inside of the existing MPAs (Fig 5D).

We performed a preliminary cost-benefit analysis between the existing MPAs in 2011 and the alternative Marxan-derived MPA (Table 3). In terms of present cost, existing MPAs required more investment (1.79 million dollars) than the Marxan-derived MPA for the MPA’s establishment and maintenance due to their larger sizes. However, this investment did not generate a higher return for the existing MPAs. Instead, Marxan-derived MPA had more present harvest revenue (0.39 million dollars) than the existing MPAs. In terms of net present values, the existing MPAs had a median value of 13.30 million dollars, while Marxan-derived MPA had a median value of 15.48 million dollars.

**Table 3:** A preliminary cost-benefit analysis between existing MPAs (2011) and Marxan MPA. Present harvest revenue and net present value show the median value with 2.5% and 97.5% quantile values in parentheses.

MPA scenarios	Present establishment cost (million dollars)	Present maintenance cost (million dollars)	Present harvest revenue (million dollars)	Net present value*(million dollars)
Existing MPAs (2011)	1.99	5.46	20.75 (17.62, 25.08)	13.30 (10.17, 17.63)
Marxan MPA	1.20	4.46	21.14 (17.49, 25.57)	15.48 (11.83, 19.91)
Difference**	+0.79	+1.00	-0.39 (-0.32, -0.49)	-2.18 (-1.66, -2.28)

*Net present value is calculated by subtracting present costs from present harvest revenue for each MPA scenario.

**Difference between existing MPAs (2011) and Marxan MPA: + indicates that existing MPAs have a great value than the Marxan MPA.

## 4. Discussion

This study presents a general framework for evaluating spatial conservation prioritization solutions in the socio-ecological system by linking conservation planning tools to the Grid-DM with biological and economic drivers. This work can be meaningful as it supports a comprehensive and structured decision-making process that consists of initial conservation planning, management strategy evaluation, and adaptive planning for tactical fisheries management. With its application in Haizhou Bay, the Grid-DM demonstrates effectiveness in capturing the spatio-temporal dynamics of the target fishery as well as the influence of fisheries conservation measures. The model provides information regarding how stock biomass changes with expanding MPAs and extended seasonal closures over time, which captures the general trend from observations. The predicted biomass distribution also presents a good consistency with the observations in most areas, suggesting that the Grid-DM, when properly tuned, can capture the spatial dynamics of a fishery.

When incorporating outcomes of spatial conservation prioritization tools in the simulation, the Grid-DM projects potential consequences of the alternative MPA. Comparing to the effects of existing MPAs in Haizhou Bay designed with little socio-economic considerations, the optimized MPA alternative solution reduces the management cost without much compromise of biological and economic benefits. Preliminary cost-benefit analysis also indicates that spatial conservation prioritization could provide a cost-effective approach for fisheries management and conservation. These findings reflect the aim of spatial conservation prioritization in balancing ecological and socio-economic objectives in the MPA design [53]. Nevertheless, its effectiveness may vary case by case. We suggest that it is still necessary to perform a systematic evaluation for those spatial conservation prioritization solutions with the proposed platform. In addition, this evaluation is intended to support, instead of replacing, stakeholder engagement throughout the decision-making process. With those evaluation metrics provided in the presented framework, we expect that they would better inform decision-makers of the trade-offs between various spatial management strategies and eventually guide them to choose the best solution for their fishery.

The hindcasting Grid-DM results (Fig 4A and 4B) showed an overestimation of biomass temporally and spatially. A slower declining rate of the simulated biomass was observed in the beginning years compared to that in the observation. This may be explained by the underestimation for natural mortality and fishing mortality rates in 2012 by interpolating the parameters in 2011 and 2013 due to the lack of data. An overestimation of biomass was also shown inside of several existing MPAs. This may be due to the zoning of these MPAs that allows recreational fishing, tourism, aquaculture, and artificial reef construction [54], while these uses were not considered in our simulation because of data constraints. In addition, existing MPAs in China have been questioned for their limited enforcement capacity and low fishermen compliance [47, 55]. Therefore, it is likely that the performance of these MPAs is overestimated in our simulation by ignoring these factors.

Overall, we see the Grid-DM as an important bridge between conservation planning and fisheries management, with several improvements made based on the existing simulation models. The general grid-based modelling platform is flexible to simulate fisheries of different natures and fish species of different life histories, which is critical for its application to a broad planning context. The model also allows evaluation for different types of MPAs, ranging from no-take reserves to multi-zone MPAs, acknowledging the diversity in conservation approaches across the globe. With this flexible platform, the model can directly use the gridded input and output files of spatial conservation prioritization tools and adaptively evaluate the effects of conservation planning solutions, which is a significant improvement on existing models used for conservation planning evaluation (e.g., [13, 34]).

Our model highlights the effectiveness of minimum realistic model for advising conservation practices and provides a less-data-needy evaluation approach for precautionary spatial management strategies. For example, in our case study of small yellow croaker in Haizhou Bay, we used the data available, including survey biomass and abundance, SRR, and migration patterns of the species, to illustrate how changing management measures may affect the fishery. In this regard, this approach can complement complex ecosystem models, such as Ecospace [14] and Atlantis [16], to address management problems for species that is data limited but has a high conservation and/or management priority.

The proposed model can be used to project temporal and spatial changes of the stock and present socio-ecological performances of management strategies. This is significant because the implementation of spatial management measures is usually associated with high economic and opportunity costs, and financial constraints have been a key challenge for conservation practices globally [56]; comprehensive evaluation is, hence, necessary to find cost-effective solutions. Comparing to traditional equilibrium models for policy screening (e.g., [29]), this model can provide more information in terms of biological and socio-economic performances at different temporal and spatial scales, and thus can improve the decision-making for fisheries management. The model has the capability in incorporating various forms of uncertainties in the simulation, which is critical in implementing a precautionary approach but fails to be explicitly addressed in many existing models (e.g., [13, 34]). In this case study, we specifically considered the uncertainties associated with SRR, fish movement and fleet dynamics. This not only better represents the characteristics in fisheries, but also enables an investigation in the effectiveness of spatial management measures with these uncertainties.

Like many other modeling approaches, Grid-DM also has some weaknesses. One is that it lacks the capability to deal with multispecies analysis, which has been advocated in a recent move towards ecosystem-based fisheries management [57]. Nevertheless, considering the interplay between tactical and strategic models, Grid-DM would likely complement other grid-based strategic ecosystem models such as Ecospace and vice versa, which would eventually contribute to a more comprehensive knowledge of management efficacy [58]. For example, by running Grid-DM in parallel with ecosystem models that consider fish community structure, decision-makers can check how the tactical advice from the model impacts other ecosystem components and whether key parameters in Grid-DM need to be adjusted in response to ecological interactions. Another limitation of the model is that it does not model the dynamics of stock in larval stages. However, this limitation can be addressed when the information on pelagic larval duration and biophysical models become available. In the present study, age or size structure were not accounted for ontogenetic movements and natural mortality which may scale allometrically with body size. We suggest such caveats should be addressed in further applications with more data becoming available.

Moving forward, we see the proposed framework and Grid-DM can be used as an important and effective platform in addressing conservation planning and fisheries management related issues. These may include comparing the efficacy and efficiency of different algorithms or spatial prioritization methods in conserving a stock, finding balances between spatial management and tradition fisheries management approaches with different fisheries management objectives, and measuring the effectiveness of spatial management strategies in a changing climate context. Furthermore, within the presented framework, we suggest that a more comprehensive sensitivity analysis should be performed to investigate the impacts of uncertainties (e.g., recruitment, natural and fishing mortality, habitat changes, and fisher behaviors) on the efficacy of conservation planning solutions in the future work. Our case study also indicates that limited enforcement capacity and fishers’ incompliance may render MPAs ineffective, which can be incorporated into the future risk analysis as well. Finally, the role of Grid-DM can be extended to serve as an operating model as a part of management strategy evaluation (MSE) [59–61]. There are some similarities between our framework and MSE: (a) both allow assessment of a range of management scenarios and thus can inform decision makers through the feedback loop; (b) stakeholders can be involved in the development and evaluation of management strategies to increase transparency in the management process and possibly compliance in the implementation process; and (c) both enable examinations of various forms of uncertainties. To this end, our model could be included in the broad MSE framework, thereby allowing the performance of Grid-DM to be further evaluated.

## Supporting information

S1 Excel Relevant data used in model development. Species biomass in each planning unit in September, 2011.
(CSV)

S1 PDF Permit of the collection sites area access by the Ocean University of China. (PDF)

S1 Image Permit of the collection sites area access by the Ministry of Agriculture of People’s Republic of China. (JPG)

S1 Table Summary of the cross-validation test for the arithmetic mean HSI model (AMM) and the geometric mean HSI model (GMM) for small yellow croaker (*Larimichthys polyactis)* in Haizhou Bay and adjacent areas based on unweighted or weighted HSI models. (DOCX)

S1 Fig The biomass distribution of small yellow croaker in September, 2011. (DOCX)

S2 Fig Relative contribution (%) of different environmental variables to the total deviance explained by the boosted regression tree (BRT) models for small yellow croaker (*Larimichthys polyactis)* in Haizhou Bay and adjacent areas. (DOCX)

S3 Fig Habitat Suitability Index of small yellow croaker in May (a) and September (b) in 2017 in Haizhou Bay.
(DOCX)

S4 Fig The result of deterministic run with calibrated parameters. (DOCX)

S5 Fig The complete figure of Fig 4B. (DOCX)

S6 Fig Comparison of observation (a) and simulation (b) in 2017.
(DOCX)

S1 Material (DOCX)

S1 File (ZIP)
