# Supplementary material for: Evaluating fisheries conservation strategies in the socio-ecological system: A grid-based dynamic model to link spatial conservation prioritization tools with tactical fisheries management
Source: PLoS One. 2020 Apr 3;15(4):e0230946. doi: 10.1371/journal.pone.0230946 (PMC7122822; doi:10.1371/journal.pone.0230946)
Supplement: S1 PDF — (PDF) [file pone.0230946.s002.pdf]

证　明

海州湾是我国黄海传统的重要渔场之一。但长期以来，气候变化及人类活动等诸多因素的影响渔业种群结构也发生了较大变化，呈现出显著的生态替代现象。该区域代表了在人类活动和气候变化等多重因素干扰下生态系统结构和功能大生重大变化的典型海湾，是研究气候变化和人类活动对渔业生态系统影响的典型区域。因此，我院自 2011 年开始以海州湾为野外观测海域，长期调查观测海州湾及邻近海域生物多样性与环境特征，在中长时间尺度上开展海州湾生态学过程和动力学机制研究，对我国近海资源修复和渔业管理具有重要意义。

中国海洋大学水产学院

2016.10
