# Supplementary figures and images for: Evaluating fisheries conservation strategies in the socio-ecological system: A grid-based dynamic model to link spatial conservation prioritization tools with tactical fisheries management
Source: PLoS One. 2020 Apr 3;15(4):e0230946. doi: 10.1371/journal.pone.0230946 (PMC7122822; doi:10.1371/journal.pone.0230946)

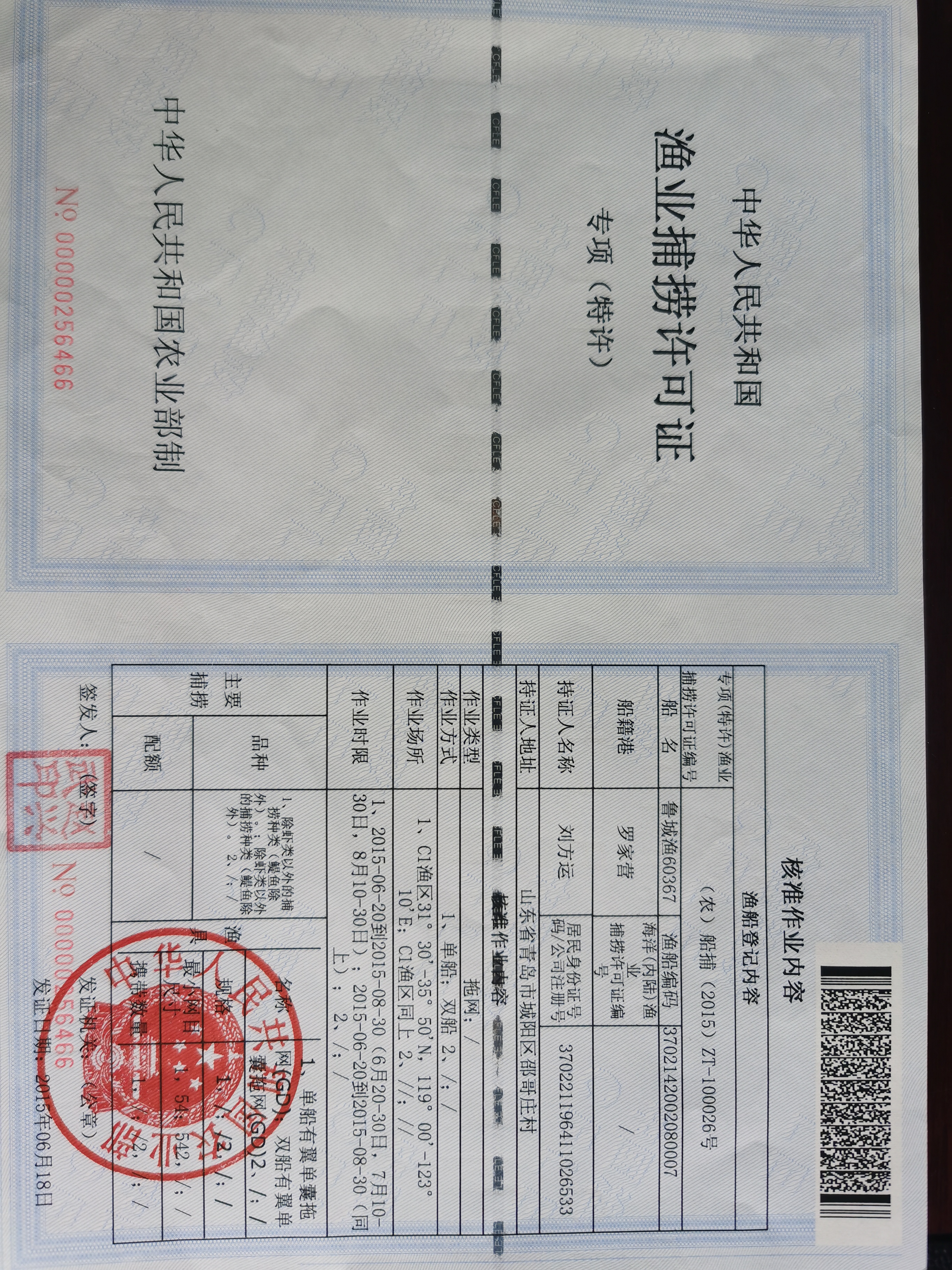

Supplement: S1 Image — (JPG) [file pone.0230946.s003.jpg]

**Fig S1**. The biomass distribution of small yellow croaker in September, 2011.

**
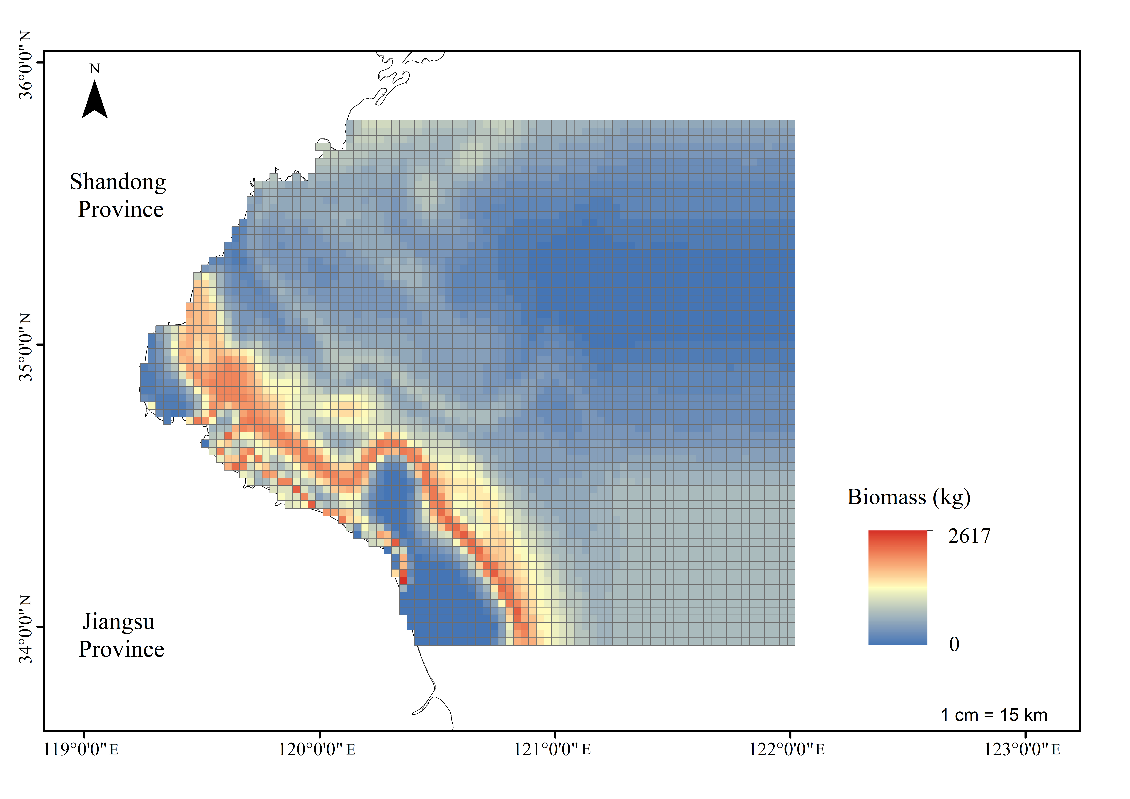
**

Supplement: S1 Fig — (DOCX) [file pone.0230946.s005.docx]

**Fig S5.** The complete figure of Fig 4b.


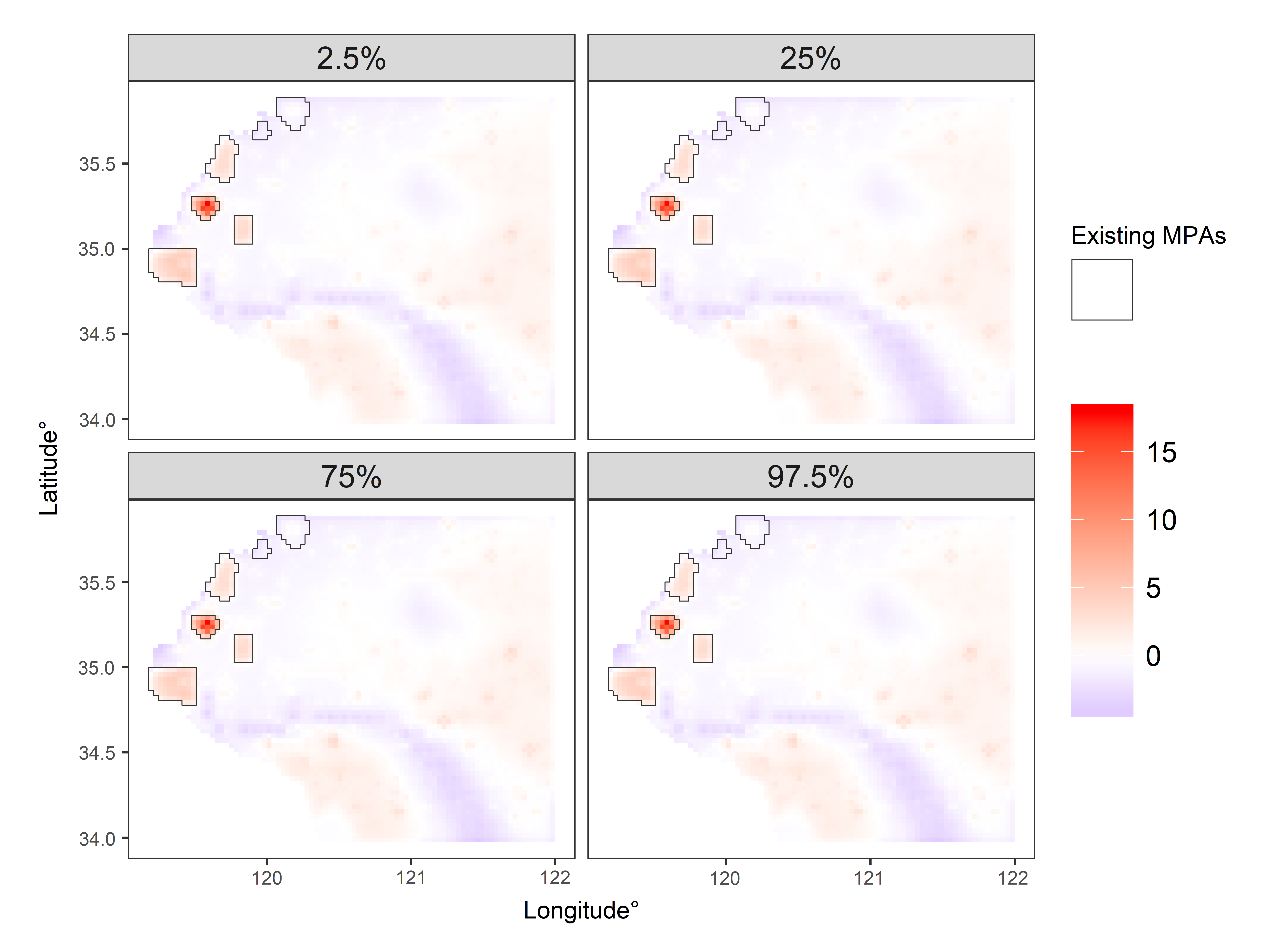

Supplement: S5 Fig — (DOCX) [file pone.0230946.s009.docx]

**Fig S6** Comparison of observation (a) and simulation (b) in 2017.


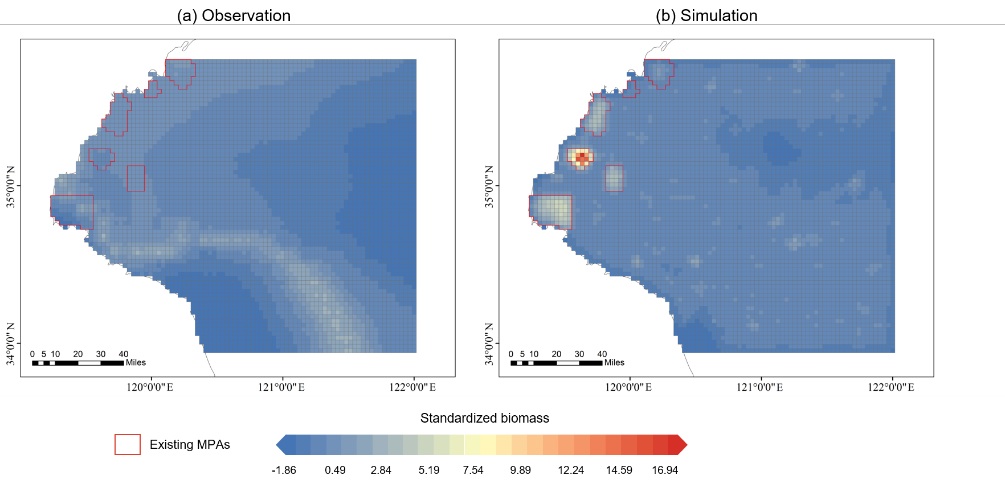

Supplement: S6 Fig — Comparison of observation (a) and simulation (b) in 2017. (DOCX) [file pone.0230946.s010.docx]
