# Supplementary material for: Evaluating fisheries conservation strategies in the socio-ecological system: A grid-based dynamic model to link spatial conservation prioritization tools with tactical fisheries management
Source: PLoS One. 2020 Apr 3;15(4):e0230946. doi: 10.1371/journal.pone.0230946 (PMC7122822; doi:10.1371/journal.pone.0230946)
Supplement: S1 Table — (DOCX) [file pone.0230946.s004.docx]

**Table S1.** Summary of the cross-validation test for the arithmetic mean HSI model (AMM) and the geometric mean HSI model (GMM) for small yellow croaker (*Larimichthys polyactis)* in Haizhou Bay and adjacent areas based on unweighted or weighted HSI models

| Seasons | Models | AMM | | | |  | GMM | | | |
| --- | --- | --- | --- | --- | --- | --- | --- | --- | --- | --- |
|  |  | Mean AIC | 95% CI | Mean R² | 95% CI |  | Mean AIC | 95% CI | Mean R² | 95% CI |
| Spring | Unweighted HSI model | 9.920 | (-1.091,20.830) | 0.433 | (0.143,0.689) |  | 13.617 | (2.691,24.026) | 0.315 | (0.070,0.568) |
|  | Weighted HSI model | 3.439 | (-7.055,14.974) | 0.566 | (0.257,0.763) |  | 12.900 | (3.246,22.929) | 0.299 | (0.033,0.629) |
| Fall | Unweighted HSI model | 21.641 | (6.456,33.476) | 0.492 | (0.135,0.769) |  | 24.818 | (10.373,34.977) | 0.405 | (0.048,0.735) |
|  | Weighted HSI model | 20.347 | (3.493,32.665) | 0.519 | (0.120,0.829) |  | 24.441 | (9.976,34.100) | 0.416 | (0.066,0.731) |
