# Supplementary material for: Evaluating fisheries conservation strategies in the socio-ecological system: A grid-based dynamic model to link spatial conservation prioritization tools with tactical fisheries management
Source: PLoS One. 2020 Apr 3;15(4):e0230946. doi: 10.1371/journal.pone.0230946 (PMC7122822; doi:10.1371/journal.pone.0230946)

**Fig S2** Relative contribution (%) of different environmental variables to the total deviance explained by the boosted regression tree (BRT) models for small yellow croaker (*Larimichthys polyactis)* in Haizhou Bay and adjacent areas.


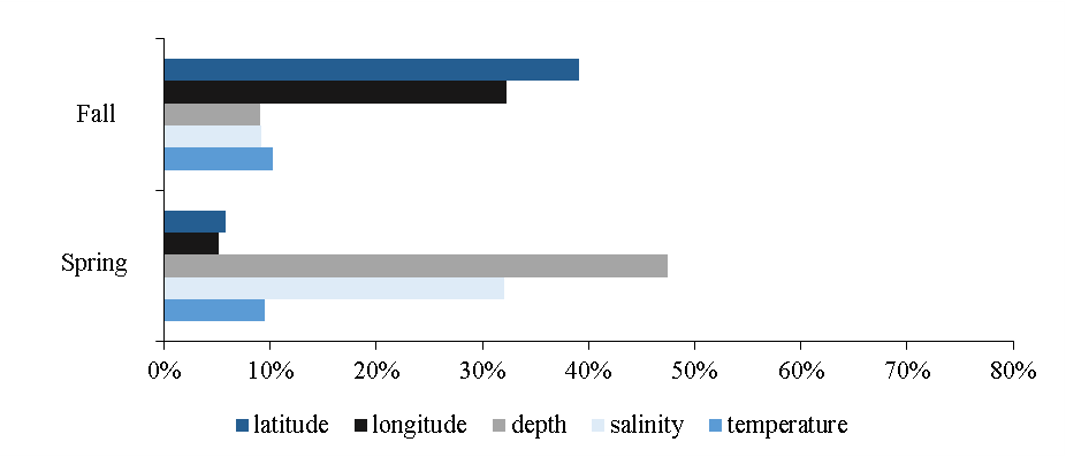

Supplement: S2 Fig — (DOCX) [file pone.0230946.s006.docx]
