# Supplementary material for: Evaluating fisheries conservation strategies in the socio-ecological system: A grid-based dynamic model to link spatial conservation prioritization tools with tactical fisheries management
Source: PLoS One. 2020 Apr 3;15(4):e0230946. doi: 10.1371/journal.pone.0230946 (PMC7122822; doi:10.1371/journal.pone.0230946)

**Fig S3**. Habitat Suitability Index of small yellow croaker in May (a) and September (b) in 2017 in Haizhou Bay.


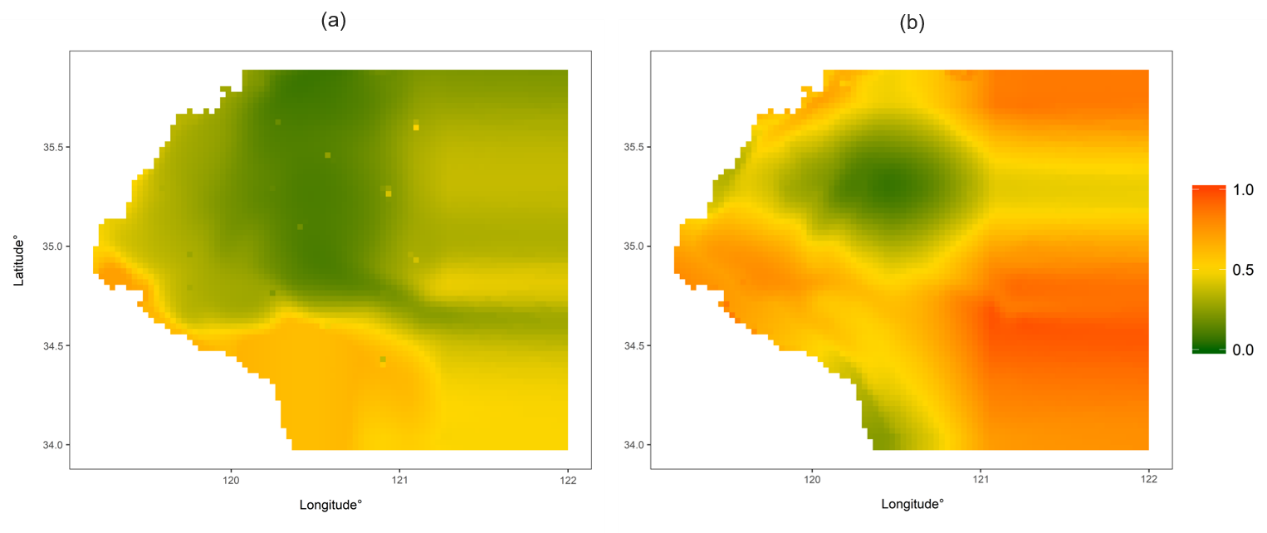

Supplement: S3 Fig — Habitat Suitability Index of small yellow croaker in May (a) and September (b) in 2017 in Haizhou Bay. (DOCX) [file pone.0230946.s007.docx]
