# Supplementary material for: Evaluating fisheries conservation strategies in the socio-ecological system: A grid-based dynamic model to link spatial conservation prioritization tools with tactical fisheries management
Source: PLoS One. 2020 Apr 3;15(4):e0230946. doi: 10.1371/journal.pone.0230946 (PMC7122822; doi:10.1371/journal.pone.0230946)

**Fig S4**. The result of deterministic run with calibrated parameters (movement rate coefficient is 0.24 and fleet aggregation factor is 1.13).


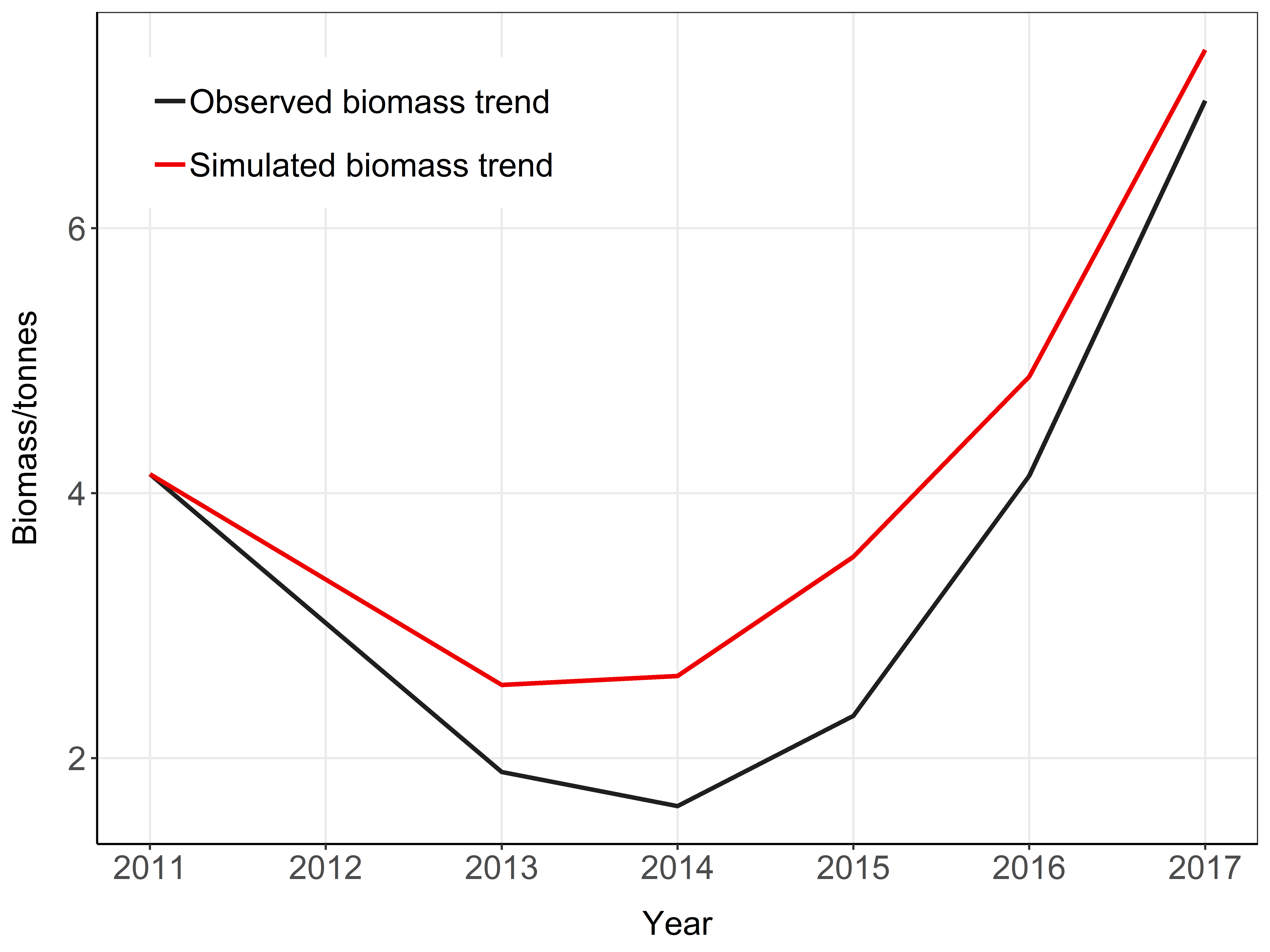

Supplement: S4 Fig — (DOCX) [file pone.0230946.s008.docx]
