# Supplementary material for: Evaluating fisheries conservation strategies in the socio-ecological system: A grid-based dynamic model to link spatial conservation prioritization tools with tactical fisheries management
Source: PLoS One. 2020 Apr 3;15(4):e0230946. doi: 10.1371/journal.pone.0230946 (PMC7122822; doi:10.1371/journal.pone.0230946)
Supplement: S1 Material — (DOCX) [file pone.0230946.s011.docx]

**Supplementary material**

Evaluating fisheries conservation strategies in the socio-ecological system: A grid-based dynamic model to link spatial conservation prioritization tools with tactical fisheries management

**Species Distribution Models**

The generalized additive model (GAM; Hastie and Tibshirani 1990) was used to quantify the statistical relationship between the biomass of small yellow croaker (*Larimichthys polyactis*) and environmental and spatial variables. The environmental and species biomass data were collected from bottom trawl surveys in Haizhou Bay and adjacent areas during fall in 2011 and 2013-2017. Surveys were designed by stratified random sampling, where a total of 24 sampling sites in 2011 and 18 sites in the following years were selected from five strata per survey (see Xu et al. 2015 for more survey details). At each survey station, a CTD system (XR-420) was used to measure environmental data including depth, bottom water temperature and salinity.

The development of GAM model was described in Li et al. 2019. The bottom water temperature (℃), salinity and latitude were used to build the GAM. We used the Finite-Volume Coastal Ocean Model (FVCOM) to project the environmental variables (bottom water temperature and salinity) over the whole study area for mapping small yellow croaker distributions. 64392 grid points were extracted from the FVCOM developed in Haizhou Bay (calibrated by College of Environmental Science and Engineering, Ocean University of China) (Luan et al. 2018). Latitude was extracted using ArcGIS 10.2 (ESRI 2014). The fitted GAM was used to map the species biomass distribution over the grid points in 2011, 2013-2017. Species biomass was then interpolated using inverse distance weighting (IDW) tool in ArcGIS 10.2 (ESRI 2014). The number of biomass in each PU was calculated using the Zonal Statistics as Table tool by ArcGIS 10.2 (ESRI 2014). The species distribution data of 2011 were used as the initial distribution data and baseline data for simulation and those of other years (2013-2017) were used as observed distribution data for calibration.

**Habitat Suitability Index**

The modelling process of Habitat Suitability Index (HSI) of small yellow croaker is based on Zou et al. 2016 with updated data in 2017. Both biomass and environmental data were collected from collected from a bottom trawl survey conducted in May and September 2011 and 2013-2017. A stratified random sampling design was used, where a total of 24 sampling sites in 2011 and 18 sites in the following years were selected from five strata per survey (Xu et al. 2015). The bottom trawl was towed at a speed of 2-3 knots for about 1 h, with a trawl net of 12m width and mesh size of 17mm. There were two bottom trawl vessels with the same size, power and trawl net, and tows were only conducted during daytime. At each survey station, a CTD system (XR-420) was used to measure environmental data including depth, bottom temperature and bottom salinity. Boosted regression trees (BRTs) were used to determine the weight of each environmental variable (bottom temperature, bottom salinity, depth, longitude and latitude) in HSI models based on their relative contributions (Figure S2). The spline smooth regression was used to fit the relationship between each environmental variable and suitability index (SI) (Chang et al. 2012). The SI values derived from each environmental variable were then combined into the empirical HSI model. Two empirical HSI models (arithmetric mean model (AMM) and geometric mean model (GMM)), which are commonly used to estimate habitat suitability, were considered:

1. AMM (Wakeley 1998)

$\mathrm{HSI}_{AMM}=\frac{1}{\sum_{i=1}^{n} w_{i}}\times\sum_{i=1}^{n} {SI}_{i}w_{i}$, (S1)

1. GMM (Vincenzi et al. 2006)

$\mathrm{HSI}_{GMM}= \left( \prod_{i=1}^{n} {SI}_{i}^{w_{i}} \right)^{\frac{1}{\sum_{i=1}^{n} w_{i}}}$, (S2)

Where *SI_i_* is the *SI* value of environmental variable *i*, *w_i_* is the weight assigned to environmental variable *i* based on BRT model outputs, and *n* is the number of environmental variables. Summary of cross-validation test for the AMM and GMM models was shown in Table S1. The value of HSI is between 0 and 1, indicating “poor” to “good” habitat quality (Figure S3).

**Movement rate**

We assumed that the basic fish movement rate in each PU is negatively related to the corresponding HSI value; therefore, a high HSI value in a PU indicates a low probability of fish movement from the PU. As the first step to calculate the basic movement rate, we fitted HSI value in each PU to a sinusoidal function of time step using open source programming language R (version 3.4.0). Then we construct the movement rate curve, which is the reflection of HSI curve over the line y=0.5, with a horizontally shift due to a time lag of one time step. The value of basic movement rate is between 0 and 1, indicating low to high probability of movement rate from each PU.

**Marine protected area planning**

We used spatial conservation prioritization tool Marxan (Ball & Possingham 2000) to identify an alternative MPA and to test whether or not the Marxan-derived MPA would outperform the existing MPAs in biological and socio-economic performances. In this comparison, we set the same protection target (percentage of species biomass included in the MPAs) in Marxan to the that for existing MPAs. Accordingly, we calculated the percentage (3.65%) of species biomass of 2011 inside of existing MPAs of 2011 using the Zonal Statistics as Table tool by ArcGIS 10.2 (ESRI 2014). Different from the selection criteria for existing MPAs which rarely considered socio-economic costs, we used the PU area as surrogate data of management costs in Marxan. Marxan was run for 100 times. Both species penalty factor (SPF) and boundary length modifier (BLM) was adjusted to ensure the solutions were adequately compact and the target was met. SPF and BLM were set as 1 and 0.005, respectively. The best solution output was adopted as an alternative MPA and was further evaluated by the Grid-DM

**Model construction**

R code for model construction be accessed on Github repository (<https://github.com/yunzhl19/Grid-PM.git>).

**Reference**

Ball IR, Possingham HP. MARXAN (V1. 8.2). Marine Reserve Design Using Spatially Explicit Annealing, a Manual; 2000.

Chang YJ, Sun CL, Chen Y, et al. Habitat suitability analysis and identification of potential fishing grounds for swordfish, Xiphias gladius, in the South Atlantic Ocean. International Journal of Remote Sensing. 2012;33(23): 7523–7541.

ESRI. ArcGIS Desktop: Release 10.2. Redlands, CA: Environmental Systems Research Institute; 2014.

Li Y, Zhang C, Xue Y, Xu B, Sun M, Ren Y, Chen Y. Developing a marine protected area network with multiple objectives in China. Aquatic Conservation: Marine and Freshwater Ecosystems. 2019;29(6): 952-963.

Luan, J., Zhang, C., Xu, B., Xue, Y., & Ren, Y. Modelling the spatial distribution of three Portunidae crabs in Haizhou Bay, China. PloS one, 2018;13(11), e0207457.

Hastie, T., Tibshirani, R., 1990. Generalized additive models (Vol. 43). CRC press.

Vincenzi S, Caramori G, Rossi R, De Leo GA. A GIS-based habitat suitability model for commercial yield estimation of Tapes philippinarum in a Mediterranean coastal lagoon (Sacca di Goro, Italy). Ecological Modelling. 2006;193(1-2):90-104.

Wakeley JS. A method to create simplified versions of existing habitat suitability index (HSI) models. Environmental Management. 1988; 12(1): 79-83.

Wood, S.N., Fast stable restricted maximum likelihood and marginal likelihood estimation of semiparametric generalized linear models. J. R. Stat. Soc. Ser. B (Statistical Methodol. 2011;73, 3–36. doi:10.1111/j.1467-9868.2010.00749.x

Xu B, Zhang C, Xue Y, Ren Y, Chen Y. Optimization of sampling effort for a fishery-independent survey with multiple goals. Environmental monitoring and assessment. 2015;187(5):252.

Zou Y, Xue Y, MA Q, Xu B, Ren Y. Spatial distribution of *Larimichthys polyactis* in Haizhou Bay based on habitat suitability index. Periodical of Ocean University of China. 2016; 46(8): 54-63. (in Chines
